# Multi-institutional retrospective analysis of learning curves on dosimetry and operation time before and after introduction of intraoperatively built custom-linked seeds in prostate brachytherapy

**DOI:** 10.1093/jrr/rrv065

**Published:** 2015-10-22

**Authors:** Hiromichi Ishiyama, Takefumi Satoh, Atsunori Yorozu, Shiro Saito, Masaaki Kataoka, Katsuyoshi Hashine, Ryuji Nakamura, Susumu Tanji, Koji Masui, Koji Okihara, Toshio Ohashi, Tetsuo Momma, Manabu Aoki, Kenta Miki, Masako Kato, Masashi Morita, Norihisa Katayama, Yasutomo Nasu, Takashi Kawanaka, Tomoharu Fukumori, Fumitaka Ito, Ryoichi Shiroki, Yuji Baba, Akito Inadome, Yasuo Yoshioka, Hitoshi Takayama, Kazushige Hayakawa

**Affiliations:** 1 Department of Radiology and Radiation Oncology, Kitasato University School of Medicine, 1-15-1 Kitasato, Sagamihara 252-0329, Japan; 2 Department of Urology, Kitasato University School of Medicine, 1-15-1 Kitasato, Sagamihara 252-0329, Japan; 3 Department of Radiology, National Hospital Organization Tokyo Medical Center, 2-5-1 Higashigaoka, Meguro-ku, Tokyo 152-8902, Japan; 4 Department of Urology, National Hospital Organization Tokyo Medical Center, 2-5-1 Higashigaoka, Meguro-ku, Tokyo 152-8902, Japan; 5 Department of Radiation Oncology, National Hospital Organization Shikoku Cancer Center, Kou-160, Minamiumemoto-Machi, Matsuyama, Ehime 791-0280, Japan; 6 Department of Urology, National Hospital Organization Shikoku Cancer Center, Kou-160, Minamiumemoto-Machi, Matsuyama, Ehime 791-0280, Japan; 7 Department of Radiology, Iwate Medical University School of Medicine, Uchimaru 19-1, Morioka 020-8505, Japan; 8 Department of Urology, Iwate Medical University School of Medicine, Uchimaru 19-1, Morioka 020-8505, Japan; 9 Department of Radiology, Kyoto Prefectural University of Medicine, 465 Kajiicho, Kawaramachi-Hirokoji, Kamigyoku, Kyoto 602-8566, Japan; 10 Department of Urology, Kyoto Prefectural University of Medicine, 465 Kajiicho, Kawaramachi-Hirokoji, Kamigyoku, Kyoto 602-8566, Japan; 11 Department of Radiology, Keio University School of Medicine, 35 Shinanomachi, Shinjuku-ku, Tokyo 160-8582, Japan; 12 Department of Urology, National Hospital Organization Saitama National Hospital, 2-1 Suwa, Wakho City, Saitama 351-0102, Japan; 13 Department of Radiology, The Jikei University School of Medicine, 3-25-8 Nishi-Shimbashi, Minato-Ku, Tokyo 105-8461, Japan; 14 Department of Urology, The Jikei University School of Medicine, 3-25-8 Nishi-Shimbashi, Minato-Ku, Tokyo 105-8461, Japan; 15 Department of Radiology, Showa University School of Medicine, Showa University Koto Toyosu Hospital, 5-1-38 Toyosu Koto-ku, Tokyo 135-0061, Japan; 16 Department of Urology, Showa University Koto Toyosu Hospital, 5-1-38 Toyosu Koto-ku, Tokyo 135-0061, Japan; 17 Department of Radiology, Okayama University School of Medicine, 2-5-1 Shikata-cho, Okayama 700-8558, Japan; 18 Department of Urology, Okayama University School of Medicine, 2-5-1 Shikata-cho, Okayama 700-8558, Japan; 19 Department of Radiology, Tokushima University School of Medicine, 3-18-15 Tokushima, Kuramoto-cho, Tokushima 770-8503, Japan; 20 Department of Urology, Tokushima University School of Medicine, 3-18-15 Tokushima, Kuramoto-cho, Tokushima 770-8503, Japan; 21 Department of Radiology, Fujita Health University School of Medicine, 1-98 Dengakugakubo, Kutsukake-cho, Toyoake, Aichi 470-1192, Japan; 22 Department of Urology, Fujita Health University School of Medicine, 1-98 Dengakugakubo, Kutsukake-cho, Toyoake, Aichi 470-1192, Japan; 23 Department of Radiology, Japanese Red Cross Kumamoto Hospital, 2-1-1 Higashiku Nagamine Minami, Kumamoto City 861-8520, Japan; 24 Department of Urology, Japanese Red Cross Kumamoto Hospital, 2-1-1 Higashiku Nagamine Minami, Kumamoto City 861-8520, Japan; 25 Department of Radiation Oncology, Osaka University Graduate School of Medicine, 2-2 Yamadaoka, Suita, Osaka 565-0871, Japan; 26 Department of Urology, Osaka University School of Medicine, 2-2 Yamadaoka, Suita, Osaka 565-0871, Japan

**Keywords:** prostate cancer, brachytherapy, low dose rate, ^125^I, intraoperatively built custom-linked seed, loose seed

## Abstract

This multi-institutional retrospective analysis examined learning curves for dosimetric parameters and operation time after introduction of intraoperatively built custom-linked (IBCL) seeds. Data from consecutive patients treated with seed implantation before and after introduction of IBCL seeds (loose seed, *n* = 428; IBCL seed, *n* = 426) were collected from 13 centers. Dose–volume histogram parameters, operation times, and seed migration rates were compared before and after introduction of IBCL seeds. At the 1-month CT analysis, no significant differences were seen in dose to 90% of prostate volume between before and after IBCL seed introduction. No learning curve for dosimetry was seen. Prostate and rectal volume receiving at least 150% of prescription dose (V150 and RV150) were higher in the loose-seed group than in the IBCL-seed group. Operation time was extended by up to 10 min when IBCL seeds were used, although there was a short learning curve of about five patients. The percentage of patients with seed migration in the IBCL-seed group was one-tenth that in the loose-seed group. Our study revealed no dosimetric demerits, no learning curve for dosimetry, and a slightly extended operation time for IBCL seeds. A significant reduction in the rate of seed migration was identified in the IBCL-seed group.

## INTRODUCTION

Both loose and stranded seeds have been recognized as standard tools for interstitial permanent brachytherapy in patients with prostate cancer. Both types of seeds were available in the USA and European countries, and brachytherapists usually have at least two options. In Japan, however, the situation has been different. Because stranded or linked seeds were not introduced until 2012, loose seeds were the only option for Japanese brachytherapists.

Zauls *et al.* first reported a push-button seed delivery system that allows the user to create intraoperatively built custom-linked (IBCL) seeds, using a combination of seeds, connectors and spacers [1]. IBCL seeds combine the benefits of loose and stranded seeds, including intraoperative customization, reduced migration and stabilization due to linking. This system was introduced to Japan in 2012, ending the 10-year monopoly of loose seeds.

Although several trials have already revealed some advantages of linked seeds, particularly in reducing seed migration [2], several concerns have been raised about disadvantages associated with this introduction, such as extended operation time and dosimetric deteriorations during the learning curve, and these concerns may make brachytherapists hesitant to introduce IBCL seeds. Do IBCL seeds show dosimetric demerits compared with loose seeds? How long is the learning curve for achieving comparable results with those obtained using loose seeds? How much would the operation time be extended?

The purpose of the present study was to analyze the learning curve for the dosimetric parameters and operation times since the introduction of IBCL seeds in 13 Japanese centers.

## MATERIALS AND METHODS

This retrospective multicenter study was conducted in Japan, and institutional review boards at each participating center approved the study protocols. Data from consecutive patients treated with seed implantation before and after the introduction of IBCL seeds were collected from the 13 centers (Table 1).

**Table 1.** Outlines of 13 participating centers

	Start of loose seeds	Start of IBCL seeds	Number of team members			Number of patients per year (2014)
			Radiation oncologist	Urologist	Medical physicist	
#1	Sept 2003	Aug 2012	3	4	0	217
#2	May 2004	Aug 2012	3	2	0	112
#3	Aug 2004	Feb 2013	2	1	0	24
#4	Dec 2004	Dec 2012	2	2	0	34
#5	Apr 2005	May 2013	2	1	0	50
#6	Jan 2007	Dec 2012	1	2	0	98
#7	Oct 2003	June 2012	3	1	0	119
#8	Jan 2005	Feb 2013	3	2	0	52
#9	Jan 2004	Oct 2012	1	1	0	21
#10	July 2004	July 2013	2	3	0	63
#11	Sept 2006	Apr 2013	4	6	0	58
#12	Jan 2007	Dec 2012	1	1	0	54
#13	April 2005	March 2013	1	1	1	11

IBCL = intraoperatively built custom-linked.

Data from a total of 854 patients (loose-seed group, *n* = 428; IBCL-seed group, *n* = 426) from 13 centers was accumulated. Seed monotherapy was used for 659 patients (loose-seed group, *n* = 347; IBCL-seed group, *n* = 312). Combination with external beam radiotherapy was used for 195 patients (loose-seed group, *n* = 81; IBCL-seed group, *n* = 114).

Two centers defined separate roles for loose seeds and IBCL seeds, using loose seeds for monotherapy and IBCL seeds for combination with external beam radiotherapy. Because intermediate or high-risk patients (National Comprehensive Cancer Network Criteria) were treated with combined therapy, significant differences in Gleason score and prescription dose were seen between loose-seed and IBCL-seed groups. A significant dosimetric difference was evident between monotherapy and combined therapy (mean dose to 90% of prostate volume (D90), 127.8% vs 121.3%, respectively; *P* < 0.05) in one of those two centers. Data from these two centers were therefore excluded from analysis of the learning curve on dosimetry and dosimetric comparison between the two types of seed. Table 2 shows patient characteristics from the remaining 11 centers.

**Table 2.** Patient characteristics

	Loose seeds	IBCL seeds	*P-value*
*n*	316	314	
Age (years)	67 (7)	69 (6)	<0.01
Initial PSA (ng/ml)	7.8 (5.0)	8.3 (6.4)	n.s.
Gleason score	6.5 (0.6)	6.6 (0.7)	n.s.
T stage			n.s.
1c	186	179	
2a	84	80	
2b	23	33	
2c	16	18	
3a	5	4	
3b	0	0	
3x	2	0	
Source activity (mCi)	0.344 (0.03)	0.349 (0.04)	n.s.
No. of seeds	72 (17)	72 (17)	n.s.
No. of needles	19.5 (3.3)	19.6 (3.3)	n.s.
Hormone therapy			n.s.
Yes	152	148	
No	164	165	
Unknown	0	1	
Prescription dose (Gy)	143.1 (13.6)	143.5 (13.2)	n.s.

Values are given as means (standard deviation) or numbers. PSA = prostate-specific antigen, IBCL = intraoperatively built custom-linked.

Patients were numbered in chronological manner in the respective centers as follows: the first, second and third patients treated with IBCL seeds were numbered as #1, #2 and #3, and so forth; the patient treated with loose seeds immediately prior to the first IBCL patient was numbered as #0, the patient before #0 was #–1, the patient before #–1 was #–2, and so forth. Patients numbered >0 were thus treated with IBCL seeds and those numbered ≤0 were treated with loose seeds. All data were consecutive and collected equally from the two types of seed. Chronological changes to D90 and operation times were assessed based on the above-mentioned patient number.

Dose–volume histograms (DVHs) were calculated for every patient using images from intraoperative ultrasonography (US) and 1 month postoperatively using computed tomography (CT). The urethra was contoured through the slices including implanted seeds or on the same slices as the prostate contouring. The rectal wall was contoured, including the sphincter muscle, through slices including implanted seeds or on the same slices as the prostate contouring. DVH parameters including D90, prostate volume receiving at least 100% dose (V100), prostate volume receiving at least 150% dose (V150), dose to 90% of urethral volume (UD90), dose to 5% of urethral volume (UD5), urethral volume receiving at least 200% dose (UV200), rectal volume receiving at least 100% dose (RV100) and rectal volume receiving at least 150% dose (RV150) were collected. Each parameter was compared between loose- and IBCL-seed groups.

In this study, seed migration was defined as a seed distant to the target (≥1.5 cm) and/or with no dosimetric contribution to the target. Plain radiography or CT was used to check for seed migration.

One-way analysis of variance was used to compare chronological changes in D90. The two-sample *t*-test was used to compare parameters between loose- and IBCL-seed groups. StatMate version 4.01 statistical software (ATMS, Tokyo, Japan) was used for data analysis. Differences were regarded as significant at the *P* < 0.05 level.

## RESULTS

### Learning curve for D90

**Fig. 1.**
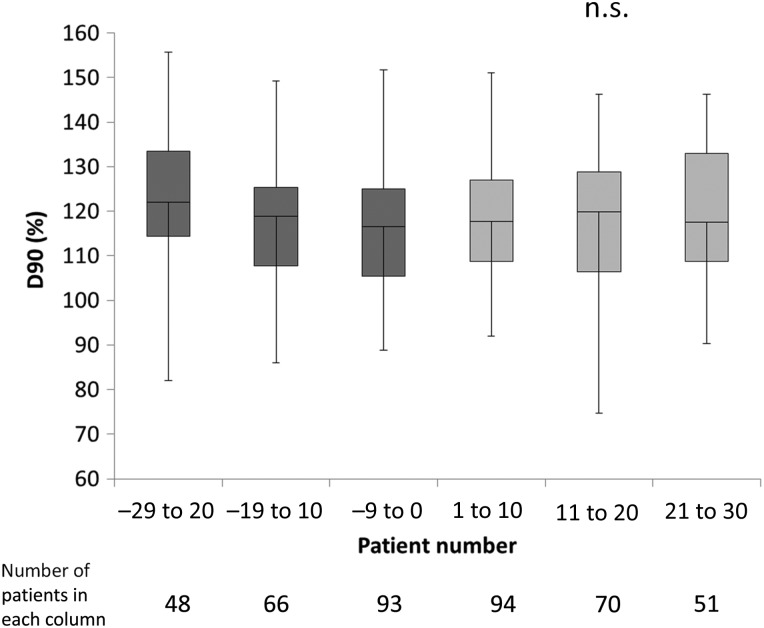
Sequential changes in D90 from the last 30 patients with loose seeds to the first 30 patients with IBCL seeds. No significant differences were seen between the six groups, consisting of equal numbers of patients with loose seeds (dark gray) and IBCL seeds (light gray). The center bar represents the median. Squares represent the 25th to 75th percentiles. Error bars indicate minimum and maximum. Data for patients ≤–30 and ≥31 were excluded because those data were only available from one center.

Figure 1 shows chronological changes to D90 from patient #–29 to patient #30. No significant differences in D90 were seen between before and after IBCL-seed introduction. Two centers, however, showed significant changes in D90 during treatment of the first 10 patients. One center showed a significant decrease in mean D90 during treatment of the first 10 patients receiving IBCL seeds (118.5%) compared with that of patients treated with loose seeds (128.3%; *P* < 0.01). These decreased values recovered to the same level as for loose seeds during the next 10 patients. The other center showed a significant increase in mean D90 (130.0%) during treatment of the first 10 patients receiving IBCL seeds compared with loose seeds (114.7%; *P* < 0.05).

### Dosimetric comparison

Table 3 shows dosimetry on US planning. The loose-seed group showed higher values for V150, UD5 and UV200 compared with the IBCL-seed group.

**Table 3.** Planning phase

	Loose seeds	IBCL seeds	*P-value*
Preope prostate volume (ml)	25.7 (3.9)	25.9 (4.1)	n.s.
Prostate volume (ml)	27.5 (8.9)	26.9 (9.2)	n.s.
Urethral volume (ml)	0.57 (0.2)	0.59 (0.3)	n.s.
Rectal volume (ml)	8.17 (3.6)	7.60 (4.6)	n.s.
D90 (%)	124.3 (13.6)	123.7 (11.9)	n.s.
V100 (%)	96.5 (3.8)	97.1 (4.4)	n.s.
V150 (%)	66.1 (14.0)	60.5 (14.8)	<0.001
UD5 (%)	157.6 (31.7)	151.4 (27.2)	0.05
UD90 (%)	99.1 (29.3)	102.4 (24.2)	n.s.
UV200 (ml)	0.0043 (0.0205)	0.0006 (0.0029)	<0.01
RV100 (ml)	0.1467 (0.2432)	0.1567 (0.2728)	n.s.
RV150 (ml)	0.0050 (0.0150)	0.0086 (0.0392)	n.s.
Operation time (min)	64.2 (20.8)	74.5 (23.7)	<0.001
Anesthesia time	89.9 (25.0)	103.4 (27.4)	<0.001

Values are given as means (standard deviation). D90 = dose to 90% of prostate volume, V100 = prostate volume receiving at least 100% of prescription dose, V150 = prostate volume receiving at least 150% of prescription dose, UD5 = dose to 5% of urethral volume, UD90 = dose to 90% of urethral volume, UV200 = urethral volume receiving at least 200% of prescription dose, RV100 = rectal volume receiving at least 100% of prescription dose, RV150 = rectal volume receiving at least 150% of prescription dose.

Table 4 shows the results for the 1-month CT analysis. Interestingly, urethral volume was slightly higher in the IBCL-seed group. V150 and RV150 were higher in the loose-seed group compared with the IBCL-seed group.

**Table 4.** One-month CT analysis

	Loose seeds	IBCL seeds	*P-value*
Prostate volume (ml)	25.7 (7.3)	26.8 (8.1)	n.s.
Urethral volume (ml)	0.48 (0.18)	0.52 (0.22)	<0.05
Rectal volume (ml)	24.6 (11.1)	25.5 (10.7)	n.s.
D90 (%)	119.3 (16.5)	118.1 (14.8)	n.s.
V100 (%)	95.5 (4.5)	95.5 (5.2)	n.s.
V150 (%)	67.6 (13.4)	60.2 (15.5)	<0.001
UD5 (%)	172.4 (38.3)	167.2 (38.5)	n.s.
UD90 (%)	105.0 (26.8)	108.2 (24.3)	n.s.
UV200 (ml)	0.0043 (0.0147)	0.0050 (0.0173)	n.s.
RV100 (ml)	0.4245 (0.0147)	0.0050 (0.0173)	n.s.
RV150 (ml)	0.0452 (0.1001)	0.0273 (0.0576)	<0.05

Values are given as means (standard deviation). Abbreviations are as in Table 3.

Differences in DVH parameters between the planning phase and 1-month CT analysis were also analyzed. For both groups, D90 and V100 tended to decrease from the planning phase to the 1-month CT analysis. Meanwhile, V150, UD5, UD90, RV100 and RV150 tended to increase from the planning phase to the 1-month CT analysis. UV200 remained stable. Table 5 shows a comparison of the two types of seeds regarding differences between the planning phase and the 1-month CT analysis. The amount of increase in V150 was significantly larger in the IBCL-seed group than in the loose-seed group (*P* < 0.05). Meanwhile, the increases in RV100 and RV150 were significantly larger in the loose-seed group than in the IBCL-seed group. Regarding UV200, the loose-seed group showed lower values than the IBCL-seed group, although the actual values were all very small.

**Table 5.** Differences between planning phase and CT analysis

	Loose seeds	IBCL seeds	*P-value*
D90 (%)	−3.14 (14.48)	−3.82 (12.86)	n.s.
V100 (%)	−2.03 (3.13)	−1.78 (4.25)	n.s.
V150 (%)	0.42 (11.91)	2.41 (14.43)	<0.05
UD5 (%)	14.69 (33.31)	17.29 (25.90)	n.s.
UD90 (%)	1.44 (23.05)	1.30 (24.92)	n.s.
UV200 (ml)	−0.0045 (0.0218)	0.0043 (0.0164)	<0.001
RV100 (ml)	0.3419 (0.4905)	0.2605 (0.4657)	<0.05
RV150 (ml)	0.0425 (0.0969)	0.0184 (0.0611)	<0.001

Values are given as means (standard deviation). Abbreviations are as in Tables 3–4.

#### Learning curve for operation time

**Fig. 2.**
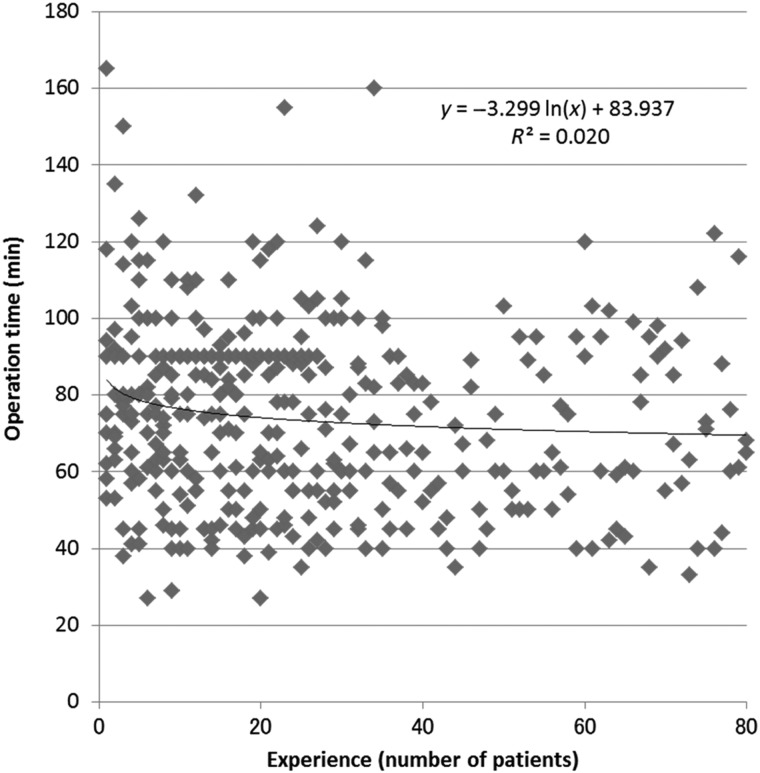
Sequential changes in operation time. The learning curve was very short, with operation time plateauing after the first five patients.

Among the 13 centers, three centers provided no data on operation time and one center had fewer than 10 patients treated with IBCL seeds. Data from nine centers were therefore used for analysis of the learning curve for operation time. Operation time was extended by as much as 10 min when IBCL seeds were used (Table 3), although the learning curve was short, at about five patients (Fig. 2).

In addition, the mean operation time of patients treated with combined therapy (73 min) was slightly longer than that of patients treated with monotherapy (68 min; *P* < 0.05).

### Seed migration

Table 6 shows seed migration rates 1 month after implantation. Data from all 13 centers were used for seed migration analysis. The percentage of patients with seed migration in the IBCL-seed group was one-tenth that in the loose-seed group. In addition, the number of migrated seeds in the IBCL-seed group was half that in the loose-seed group.

**Table 6.** Seed migration rates 1 month after implantation

	Loose seeds	IBCL seeds	*P-value*
Patients with seed migration			
Lungs			<0.001
Yes	39 (9.1%)	1 (0.2%)	
No	340 (79.4%)	385 (90.0%)	
Unknown	49 (11.4%)	40 (9.3%)	
No. of migrated seeds in patient	1.51 (0.68)	1 na	
Abdomino-pelvis			<0.001
Yes	77 (18.0%)	8 (1.9%)	
No	306 (71.5%)	378 (88.3%)	
Unknown	45 (10.5%)	40 (9.3%)	
No. of migrated seeds in patient	1.68 (1.02)	1 (0.00)	
Total			<0.001
Yes	98 (22.9%)	9 (2.1%)	
No	281 (65.7%)	377 (88.1%)	
Unknown	49 (11.4%)	40 (9.3%)	
No. of migrated seeds in patient	1.92 (1.24)	1 (0.00)	

Values are given as means (percentage or standard deviation). IBCL = intraoperatively built custom-linked.

## DISCUSSION

Our study revealed no significant change in D90 between before and after the introduction of IBCL seeds. This result would encourage brachytherapists who use only loose seeds to consider IBCL seeds as a feasible option.

Although most centers did not show significant changes to D90, our study revealed one center showing decreased D90 and another showing increased D90 during treatment of the first 10 patients after introducing IBCL seeds. The former center routinely used high D90 (128.3%) and the latter used a relatively low D90 (114.7%) using loose seeds. Centers that use high D90 in routine practice may thus show a learning curve for D90.

Because our analysis was retrospective and included the learning curve process, some biases exist in the comparison between IBCL- and loose-seed groups. One prospective analysis from a single center has already reported no dosimetric difference between loose and IBCL seeds [2]. The present multi-institutional study, however, offers higher statistical power compared with previous papers because of the relatively large number of patients. Our study thus revealed a small but significant difference between the two types of seeds.

Regarding planning phase dosimetry (Table 3), small but significant differences in V150, UD5 and UV200 were identified between loose- and IBCL-seed groups. These three parameters correlated with each other and were increased when seeds were implanted close to each other. The distance between IBCL seeds was unchanged because of the use of spacers, but distance between loose seeds was variable and perhaps tended to be smaller compared with IBCL seeds. In addition, some physicians implanted the seeds inside the prostate very carefully to avoid migration when they used loose seeds, but might not have when they used IBCL seeds. Such mechanical and technical differences may have made some contribution to the differences in V150, UD5 and UV200 in the planning phase.

On CT analysis 1 month after treatment, differences in UD5 and UV200 had disappeared, although the difference in V150 remained (Table 4). Because no urethral catheter was placed for CT analysis in most centers, detecting small differences in urethral dosimetry was difficult.

From a clinical perspective, whether the 7% difference in V150 would exert a significant impact on toxicity is unclear. Keyes *et al.*, however, reported V150 as a significant risk factor for Grade ≥2 late urinary toxicity after seed implantation [3]. They reported an odds ratio of 1.02 per 1% increase in V150. An increase of 7% with loose seeds may thus result in a 15% increase in late urinary toxicity. This may represent an advantage of IBCL seeds, although long-term toxicity was not assessed in the present analysis.

Another interesting point was the difference in urethral volume between IBCL and loose seeds (Table 4). A difference in volume of 0.04 ml (40 mm^3^) means a difference in length of 4 mm, on the assumption that the cross-sectional area of the urethra on each CT slice was ∼10 mm^2^ (the area of a triangle with sides of 5 mm). Meanwhile, the prostate volume was no different between groups. Our results therefore suggest that IBCL seeds make the prostate keep a ‘long and unnaturally straight’ shape by supporting the prostate like a bony framework.

IBCL seeds may be useful for decreasing rectal dose, although the actual value measured was small. Table 5 shows the increase in rectal dose from US planning to CT analysis, with this occurring for both types of seed. This phenomenon has already been reported in our previous paper [4]. However, the amounts of increase for RV100 and RV150 were significantly larger in the loose-seed group than in the IBCL-seed group. Although the reason for this was unclear, IBCL seeds might maintain an unnaturally straightened prostate shape and keep the prostate away from the rectal wall.

Regarding operation time, ∼10 min more was needed when IBCL seeds were used (Table 3). Although a short learning curve of about five patients was seen, the reduction rate was small (Fig. 2: approximation formula). The approximation formula suggested that operation time would be reduced by 2.3 min, 1.3 min, 1.0 min, 0.7 min and 0.6 min after experience of 1, 2, 3, 4 and 5 patients, respectively. There is no choice but to spend the time needed to build up seeds and spacers during operations when IBCL seeds are used. We believe, however, that an increase of 10 min is not problematic for most patients and physicians.

Several papers have already reported on seed migration for loose seeds and strand or linked seeds [5–10]. Our results are supported by many previous studies that have also shown significant reductions in seed migration rates for stranded seeds, including IBCL. Although the clinical impact of seed migration is debatable, IBCL seeds can resolve some of the anxieties about significant but very rare adverse events such as radiation pneumonitis [11], acute myocardial infarction [12] and small-cell lung cancer [13] associated with seed migration.

Some limitations must be considered when interpreting the results of the present study. Because this study was a retrospective analysis based on medical records, some biases might exist, especially in dosimetric comparisons. In addition, because most of the centers included in this study were high-volume centers such as university hospitals or national centers, our results may not be applicable to low-volume centers such as community hospitals.

In conclusion, this multi-institutional retrospective study revealed no dosimetric demerits, no learning curve on dosimetry, and a slightly extended operation time for IBCL seeds. Meanwhile, a significant reduction in the rate of seed migration was shown in the IBCL-seed group.

## CONFLICT OF INTEREST

Dr Ishiyama reports grants from Medicon Co. Ltd and Nihon Medi-Physics Co. Ltd during the conduct of the study, and personal fees from Medicon Co. Ltd and Nihon Medi-Physics Co. Ltd outside the period of the submitted work. Dr Satoh reports grants from Daiichi Sankyo Co. Ltd, Astra Zeneca, Janssen Pharmaceutical K.K. and Astellas Pharma Inc. outside the period of the submitted work, and honoraria for educational lectures. Dr Hayakawa reports grants from Medicon Co. Ltd and Nihon Medi-Physics Co. Ltd during the conduct of the study.
